# Pathological Perspective of Drug-Eluting Stent Thrombosis

**DOI:** 10.1155/2012/219389

**Published:** 2012-05-14

**Authors:** Katsumi Inoue

**Affiliations:** Department of Laboratory Medicine, Kokura Memorial Hospital, 3-2-1 Asano, Kokurakita-ku, Kitakyushu, Kokura 802-8555, Japan

## Abstract

Although very late stent thrombosis (VLST) after drug-eluting stent (DES) implantation remains a major concern, the precise mechanisms have not been clarified. An association between late acquired incomplete stent apposition (ISA) and VLST of DES has been suggested by several intravascular ultrasound studies demonstrating very high prevalence of ISA in the setting of VLST. To clarify the pathological mechanisms of VLST, we investigated vascular responses of coronary arteries of VLST cases after DES implantation.

## 1. Introduction

Drug-eluting stents (DESs) have dramatically reduced angiographic restenosis and clinical need for repeat revascularization procedures [1, 2]. However, concerns have been raised about the safety of DES, and certain issues remain unsolved. One of the most important issues raised is stent thrombosis (ST) [3], a catastrophic, albeit infrequent, complication that results in abrupt coronary artery closure, which can lead to myocardial infarction or sudden cardiac death [4]. ST can occur with either bare-metal stents (BMSs) or DES [5]. Acute or subacute ST includes the events occurring either during the index procedure (acute) or within 30 days (subacute). Late ST (LST) has been defined as occurring from 30 to 360 days after the procedure. Very late ST (VLST) has been defined as occurring >1 year later [6]. LST and/or VLST has emerged as a distinct entity overshadowing the use of DES, and concerns persist as to whether this phenomenon might jeopardize the long-term outcome after DES implantation [7]. Furthermore, long-term follow-up studies revealed that VLST could occur at a rate of 0.1% per year even in patients with BMS implantation, although the annual incidence of VLST of BMS was much lower than that after DES implantation [8]. I describe here the mechanism of ST, especially LST and VLST of DES, from a pathological standpoint. 

## 2. Mechanisms of ST in DES

### 2.1. Delayed Arterial Healing

**Figure 1:**
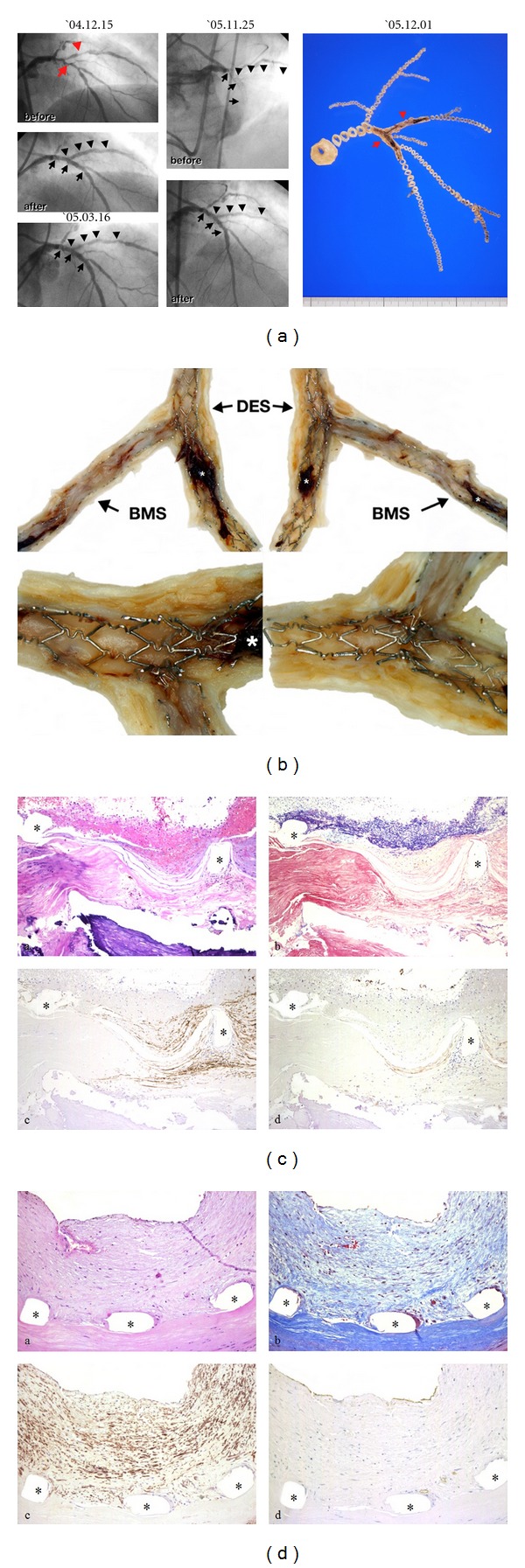
(a) The left panels show angiograms of a stable angina patient who underwent successful stent implantation in the native left anterior descending coronary artery with SES and the first diagonal branch with BMS. More than 11 months after stenting, severe thrombotic occlusion occurred at the proximal SES site, 1 week after cessation of antiplatelet medication. Despite complete revascularization by thrombus aspiration, the patient died of multiorgan failure 1 week later. (b) Macroscopically, neointimal coverage of the SES struts was scarcely visible (*blood clot formed at agonal stage). In contrast, complete coverage by neointima was observed at the BMS site. (c) Microscopic observation demonstrated no obvious endothelialized struts at the SES site, even at the portion where a mild proliferative response of smooth muscle cells was evident. At the luminal surface of these nonendothelialized struts, the remnants of fibrin-rich thrombus were visible. (d) In the BMS segment, however, relatively large neointimal coverage of stent struts was observed. Furthermore, complete re-endothelialization was clearly visible.

 The pathological findings from patients who died of late DES thrombosis have demonstrated that delayed arterial healing characterized by incomplete reendothelialization is an important underlying factor [9]. The pathologic findings of our study revealed incomplete reendothelialization and sparse smooth muscle cell coverage compared with that with BMS implanted for a similar duration (Figure 1). Although the drugs of DES, such as sirolimus and paclitaxel, reduce neointimal formation by impending smooth muscle cell migration and proliferation, these drugs can also impair the normal healing process of the endothelial cells in injured arterial wall [10]. Thus, LST may be more frequently related to incomplete healing and/or inadequate neointimal coverage. 

### 2.2. Late Acquired Stent Malapposition

**Figure 2:**
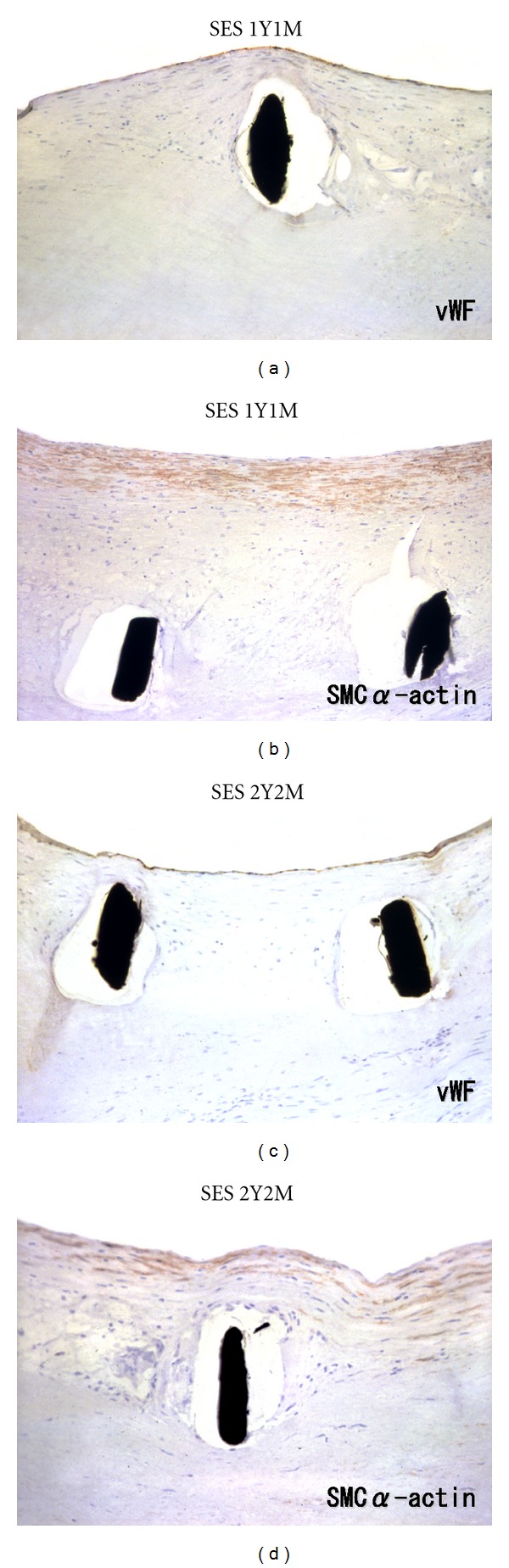
Histological sections of the SES segments harvested beyond 1 year (upper panels) and 2 years (lower panels) after stenting. Note complete neointimal coverage composed of smooth muscle cells with obvious reendothelialization.

Although a correlation has been observed between uncovered DES struts and LST, in our pathological studies of Japanese patients, considerable number of cases showed neointimal coverage with reendothelialization of a great deal of the DES struts, especially in simple lesions beyond 1 year (Figure 2). Thus, endoluminal mural thrombus in VLST cases may be present despite neointimal coverage and may reflect underlying unusual vessel responses to DES, such as late acquired incomplete stent apposition (ISA). Late ISA is highly prevalent in patients with VLST after DES implantation [11]. We have revealed two major representative pathological features concerning LSA: (1) medial necrosis and (2) peri-stent contrast staining and aneurysm formation after DES implantation. 

#### 2.2.1. Medial Necrosis

**Figure 3:**
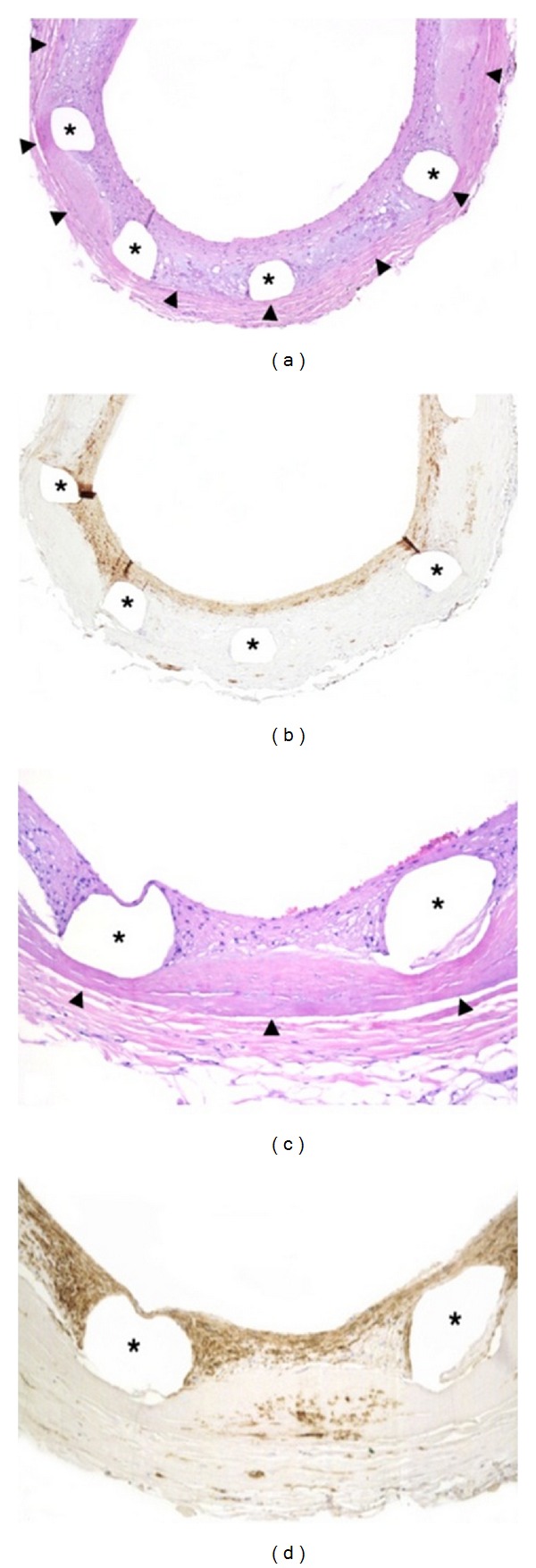
Histological sections of the plaque-free regions with SES implantation after 11.5 months. Note remarkable depletion of medial smooth muscle cells (arrowheads) just underneath the SES struts (*).

 Late ISA has been observed on follow-up intravascular ultrasound (IVUS) in patients who received sirolimus-eluting stents (SESs) [12]. Although the precise mechanism of late ISA has not been clarified, focal positive vessel remodeling is thought to be a potent candidate. We have reported an autopsy case showing medial necrosis at the segment of SES several months after implantation [13]. It is suggested that cytostatic effects of sirolimus on neointimal formation could be complicated by these local cytotoxic effects followed by a decrease in arterial tension, causing stent malapposition. The IVUS study investigating ISA portions also showed that late acquired ISA in SES was mainly present on relatively disease-free sites of the vessel wall [12]. Interestingly, we observed that medial smooth muscle cell depletion was present only in the portions where SES struts directly contacted the medial wall layers (Figure 3). We can deduce that diffusion of sirolimus from SES to the medial layer might be blocked if atherosclerotic plaques are positioned between SES struts and the medial layer.

 Thus, late ISA by focal positive vessel remodeling caused by medial necrosis may be responsible for LST and/or VLST after DES implantation.

#### 2.2.2. Peri-Stent Contrast Staining and Coronary Aneurysm Formation

**Figure 4:**
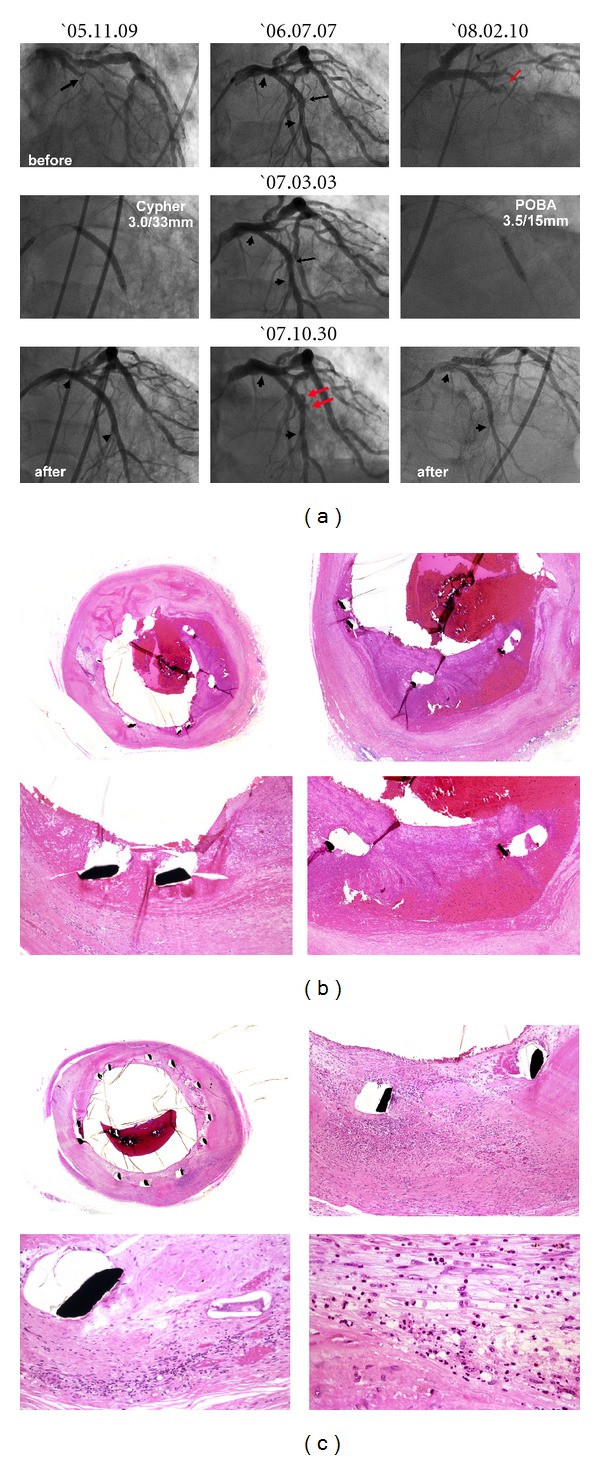
(a) These angiograms demonstrated that the SES stented site showed a tendency toward irregularly shaped coronary ectasia (peri-stent contrast staining; PSS) and finally saccular aneurysm formation 2 years after SES implantation, followed by VLST 3 months later. (b) Pathological examination demonstrated focal strut malapposition with aneurysmal dilatation and partially occlusive mural thrombus. (c) In addition, extensive inflammation, consisting primarily of lymphocytes and eosinophils, with a focal giant cell reaction, was evident at the stented site. An asteroid body with intense foreign body granulomatous inflammation was also visible (arrow). Such localized hypersensitivity vasculitis existed primarily around the struts and extended to the intima and adjoining media and adventitia.

 Peri-stent contrast staining (PSS) was defined as contrast staining outside the stent contour extending to ≥20% of stent diameter measured by quantitative coronary angiography. PSS found within 12 months after SES implantation appeared to be associated with subsequent VLST [14]. PSS could be regarded as representing an abnormal vessel wall response to DES. Coronary aneurysm and a mild form of PSS could be regarded as a continuum of the vessel wall pathological process at the site of DES implantation. We have reported the first case with VLST demonstrating serial changes in contrast staining outside the stent border leading to aneurysm formation as well as histopathologic evidence of hypersensitivity vasculitis in the stented segment (Figure 4) [15]. Virmani et al. [16] also demonstrated localized hypersensitivity vasculitis of the arterial wall within the stented segment in a patient who died of VLST. These two pathological cases suggest that chronic inflammation and/or hypersensitivity vasculitis to a polymer (a constituent component of DES) might be an important underlying mechanism of PSS and coronary aneurysm. Furthermore, these pathological findings demonstrated that inflammatory cells diffusely infiltrated the media, causing medial disruption and destruction, which might result in loss of elastic integrity of the vessel wall leading to ISA. Thus, resultant inflammation alters the vessel wall structures, causing positive remodeling. Although relatively mild inflammatory changes are recognized as PSS on coronary angiography, extensive vessel wall destruction by severe inflammation can lead to aneurysm formation in extreme cases.

### 2.3. Exaggerated Neoatherosclerosis and Neointimal Disruption following DES Implantation

**Figure 5:**
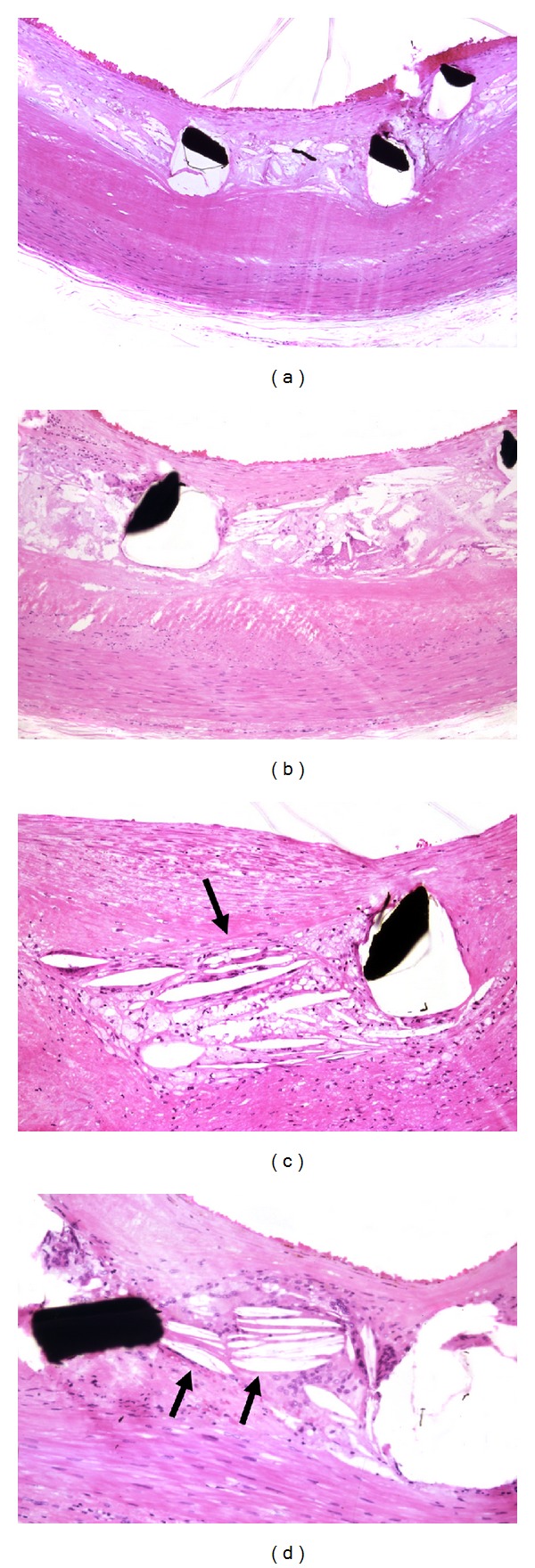
Histological sections from a patient with SES implantation 2 years antemortem. Note early necrotic core formation with pronounced foamy macrophages and circumferential cholesterol clefts (arrows) around the struts.

**Figure 6:**
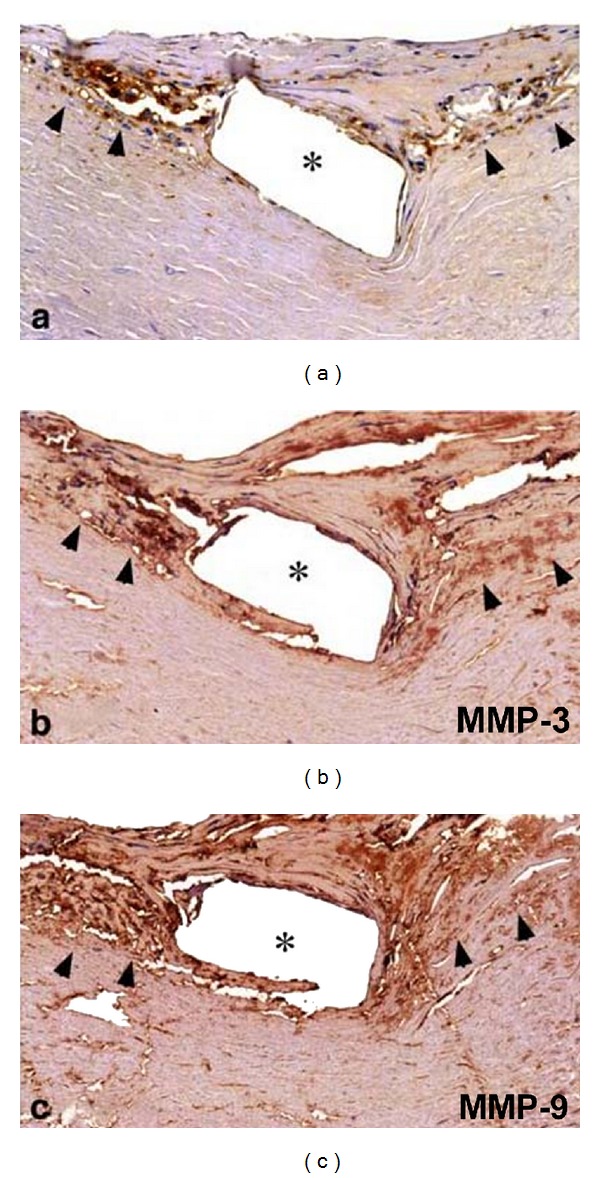
These foamy macrophages (arrowheads) clearly showed immunoreactivity to CD68, MMP-3, and MMP-9. Positive staining for these MMPs was also observed in the extracellular space.

**Figure 7:**
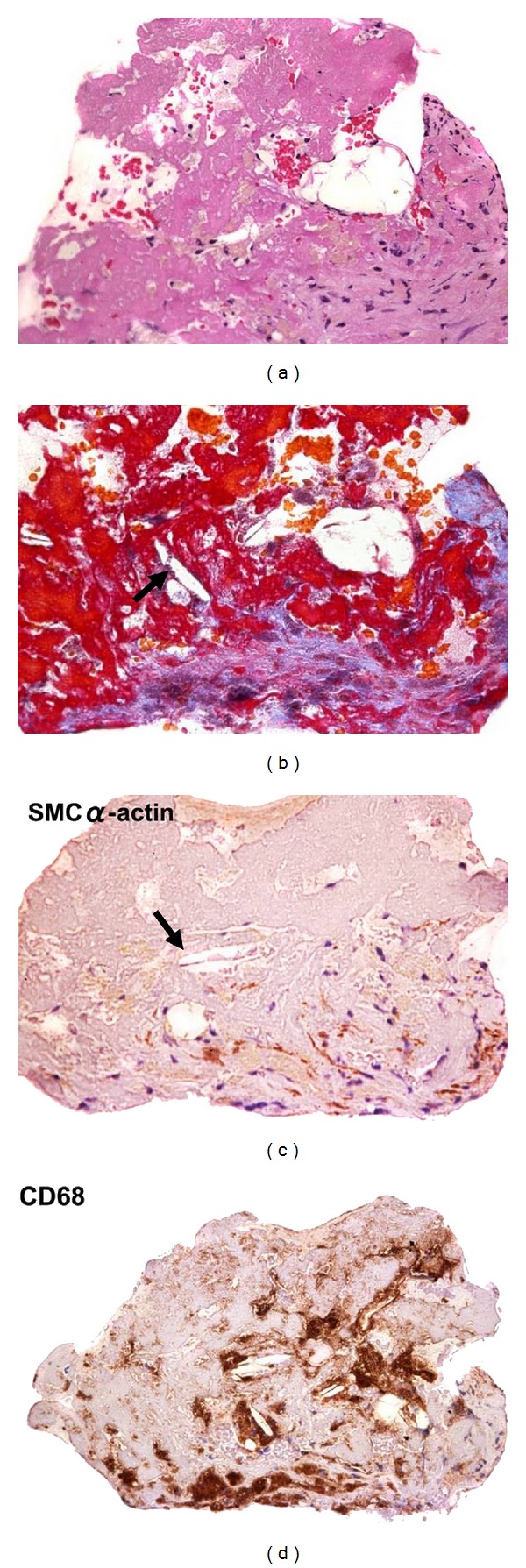
Micrographs of an aspirated specimen from the VLST site in the DES-implanted segment. Note the intimal disruption and adherent thrombus. Numerous macrophages and cholesterol clefts (arrows) were also visible in both the neointima and the thrombus.

Recent studies have identified immune cells and mediators at work in atheroma, implicating inflammatory mechanisms in disease development [17]. As previously described, in DES-implanted segments, inflammation against the durable polymer of the DES, especially heavy infiltration of macrophages around the struts, is typical. Furthermore, the remarkable presence of lipid-laden foamy macrophage infiltration within the neointima is usually evident more than several months after DES implantation. In addition, we [18] and others [19] have demonstrated that extracellular lipid, such as cholesterol crystals, accumulates and early necrotic core formation is frequently observed more than 1 year after DES stenting (Figure 5). Recent angioscopy studies have also revealed that DES promoted the formation of atherosclerotic yellow neointima in the stent-implanted lesion at 10-month follow-up [20]. Furthermore, even in the BMS-implanted segments, it has been reported that heavy infiltration of such macrophages around the struts implanted for more than 4 to 5 years was documented. These lipid-laden macrophages showed collagen-degrading matrix metalloproteinase immunoreactivity, which can degrade the neointimal layer (Figure 6) [21], followed by disruption of the stented portion (Figure 7). Recently, we examined thrombectomy specimens from a series of 135 patients undergoing angiography and thrombectomy for definite ST [22]. Fragments of atherosclerotic plaques, including foamy macrophages, cholesterol crystals, and thin fibrous caps, were observed more commonly in the extracted thrombi (under negative pressure) from cases of early ST and VLST, beyond 3 years, as opposed to LST, and were similar to what was retrieved in acute coronary syndrome. These results suggest that disruption of neoatherosclerotic neointima may be an important background for very late thrombotic events after both DES and BMS placement.

Thus, DES can induce atherosclerotic and thrombogenic lesions with a significantly higher incidence and earlier than with BMS. 

## 3. Conclusion

 LST may be more frequently related to incomplete healing and/or inadequate neointimal coverage with poor re-endothelialization. However, in the cases of VLST, several pathological studies have suggested a causal relationship between the inflammatory responses to the durable polymer and VLST, provoking late ISA and accelerated atherosclerosis followed by neointimal disruption. Histopathologic differences (the prevalence of eosinophils, giant cells, and fibrin) among DES platforms have been observed; this may reflect unique responses to the specific polymer/drug.

 Thus, until novel DES using superbly biocompatible and/or biodegradable polymers becomes available, we still need to be cautious and carefully keep surveying these devices.
